# The role of pathogen‐mediated insect superabundance in the East African emergence of a plant virus

**DOI:** 10.1111/1365-2745.13854

**Published:** 2022-03-13

**Authors:** Ruairí Donnelly, Christopher A. Gilligan

**Affiliations:** ^1^ Department of Plant Sciences University of Cambridge Cambridge UK

**Keywords:** invasion, manipulation, vector, superabundance, plant pathogen

## Abstract

1. One of the major crops for food security is cassava. Superabundant *Bemisia tabaci* whitefly, comprising unusually high landscape populations of the insect, have been implicated in cassava virus emergence. Studies have been unable to select from several hypotheses, however, as to the dynamic drivers of superabundant whitefly associated with the emergence in East Africa of severe cassava mosaic disease. One possibility is that pathogenic modification of infected plants can itself increase the growth of insect vector colonies on infected plants.
2. Through the modelling of population processes at the landscape scale we introduce a framework for analysing patterns in the association of disease and insect waves.
3. Our analyses demonstrate the role of pathogen‐mediated insect superabundance in a plant disease invasion.
4. *Synthesis*. An elevated abundance of insects at the landscape scale is frequently implicated in invasions of the plant pathogens that they carry. We advance ecological understanding of plant disease invasions by showing how landscape data can be used to investigate the causes of insect vector superabundance.

## Graphical abstract

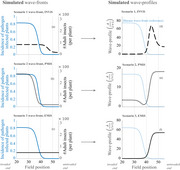

## 1 INTRODUCTION

An elevated abundance of insect vectors at the scale of individual plants and at the landscape scale is frequently implicated in the landscape invasion of insect‐borne plant pathogens. Here, and in a preceding paper (Donnelly & Gilligan, 2020), we refer to this phenomenon as insect superabundance to signify high incidence at both the individual plant scale as well as the landscape scale (note that some authors prefer to refer to this phenomenon using the terminology of ‘insect outbreak’, or, simply ‘high insect abundance’). Superabundance may simply reflect high environmental suitability for the vector (henceforth we call this scenario environment‐mediated insect superabundance, *EMiS*). An alternative cause of superabundance is the invasion of a novel insect vector strain capable of reaching higher abundance than the previously dominant strain (henceforth we call this scenario invasive vector insect superabundance, *INViS*). An additional cause of superabundance, however, that is independent of environmental suitability and insect invasion, is *PMiS* (denoting pathogen‐mediated insect superabundance), in which pathogen infection improves the resource quality of plants for insect vector multiplication [see Supporting Information S1 for the quantitative definition of pathogen‐mediated insect superabundance (PMiS) from Donnelly & Gilligan, 2020]. Since, in each scenario the landscape emergence of the plant pathogen occurs together with superabundance, a practical question then follows. How can we identify the causes of insect vector superabundance in order to determine the dynamic drivers of disease invasions? In this paper we introduce a method for distinguishing PMiS and INViS from EMiS in landscape data. Our main goal is to use this method to investigate the dynamic drivers of the East African emergence of severe cassava mosaic disease.

The density of *Bemisia tabaci* whitefly across east and central sub‐Saharan Africa has increased since the 1990s, from a few adults to hundreds per cassava shoot tip (Legg et al., 2006). The change in abundance has been associated with regional epidemic spread of cassava mosaic geminiviruses (CMGs) that they vector (Colvin et al., 2004). Specifically, there has been a simultaneous expansion of *B. tabaci* superabundance and severe cassava mosaic disease (CMD), which in turn has been linked to co‐infection of CMGs and in particular *East African cassava mosaic virus‐Uganda* (EACMV‐Ug; Colvin et al., 2006). Two principal hypotheses have been advanced to explain *B. tabaci* superabundance, first, genetic changes in the *B. tabaci* population itself (Legg et al., 2002; cf. INViS). Second, regionally, the environment has become more suitable to whitefly multiplication (cf. EMiS). In addition, a third possibility is a synergistic interaction between CMG‐infected cassava plants and *B. tabaci* (Colvin et al., 2004; cf. PMiS). To date none have been definitively proven but recent studies have appeared to diminish the role of PMiS (Boni et al., 2017). It should be noted, however, that additional factors such as host cultivar are also likely to contribute to high insect abundance (these and other factors have been reviewed in Macfadyen et al. (2018) and Macfadyen et al. (2021)). Note also that *B. tabaci* is a species complex and the evidence for INViS will later be discussed in terms of the subtypes within this complex (Discussion section). For the purposes of this paper the species complex, and the virus coinfection, are of secondary importance. For simplicity, we henceforth refer to the disease as CMD, with causative agent CMG, and insect vector *B. tabaci* (while bearing in mind that our results need not apply for CMGs beyond EACMV‐Ug and East Africa).

It is increasingly recognised that pathogen infection of plants can alter the host environment for the insect vector (Colvin et al., 2004). This effect is sometimes referred to as pathogen manipulation (Carr et al., 2018) or pathogen modification (Donnelly et al., 2019). For instance, it has been found that cassava plants infected with CMG have higher whitefly abundance than healthy cassava plants. In addition, this increased abundance has been linked to high amino‐acid concentrations in virus‐infected phloem (Colvin et al., 2006). While these observations make a role for the pathogen in insect superabundance feasible, high abundance on individual infected plants alone need not lead to landscape superabundance (Donnelly & Gilligan, 2020). If the incidence of pathogen‐infected plants is additionally high, however, then landscape superabundance can occur (Donnelly & Gilligan, 2020). As such, PMiS is an emergent property at the landscape scale—with the corollary that it must be sought in landscape data rather than in data at the scale of local plant populations. Suitable landscape datasets to identify mechanisms for superabundance should therefore include observations of both pathogen incidence and vector abundance. The datasets should also encompass a spatial gradient in the incidence of pathogen‐infected plants—as is the case for two important studies of CMG emergence in East Africa: Colvin et al. (2004)'s landscape experiment and Legg and Ogwal (1998)'s landscape survey.

Our main objective in the current paper is to infer the role of a key dynamic driver of cassava mosaic disease expansion in East Africa. To achieve this, we introduce a framework that implements population processes at the landscape scale. The framework distinguishes three superabundance scenarios that represent hypotheses as to the cause of insect vector superabundance. The scenarios are pathogen‐ (PMiS), vector‐ (INViS) and environment‐driven (EMiS) superabundance. The framework encodes the scenarios within a landscape simulation model. Each scenario generates qualitatively similar plant disease wave fronts that spread across landscapes. But analysis of the associated ‘wave profiles’ (characterised by the spatial gradient in the ratio of insect abundance to number of infected plants per field) reveal qualitatively distinct patterns characteristic of each scenario. A simple statistical analysis of wave profile patterns, capable of distinguishing among the scenarios, then follows. We use the statistical analysis to analyse an experimental dataset (Colvin et al., 2004), and, separately, a dataset resulting from a landscape survey (Legg & Ogwal, 1998), to produce new evidence, at the landscape scale, suggesting a role for PMiS in the expansion of severe CMD.

## 2 MATERIALS AND METHODS

**FIGURE 1:**
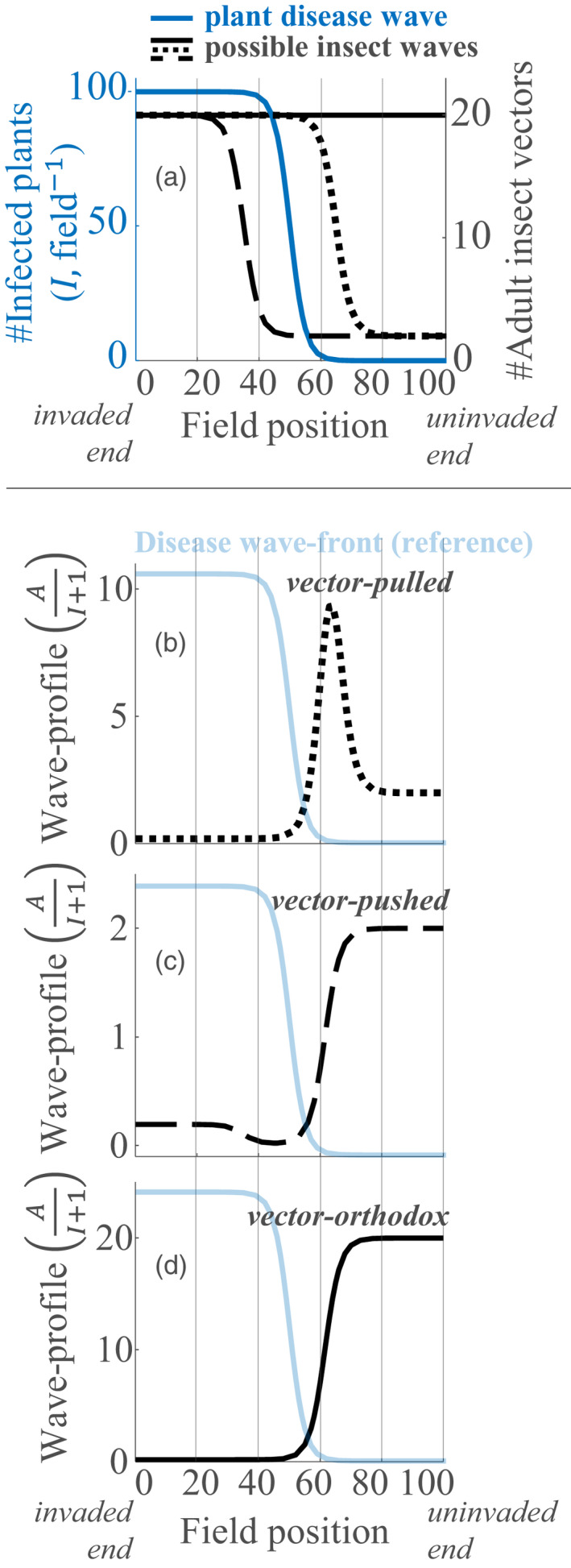
Three possible relations between plant disease wave fronts and insect abundance produce characteristic spatial patterns. In (a) representative insect wave fronts are shown either in advance of (black dotted curve), or, in the wake of (black dashed curve), a representative disease wave front (blue curve), alternatively, insect abundance may be high across the landscape (black solid curve). The spatial patterns that arise from the three relationships (a) are shown in b–d: A peaked wave profile characterises a vector‐pulled disease wave (b); a dipping wave profile characterises a vector‐pushed disease wave (c); a monotonically increasing wave profile characterises a vector‐orthodox disease wave (d). See Box 1, for detailed description and summary. Disease and abundance wave fronts (a) were generated from logistic curves. Wave profiles (b–d), that is, the ratio of insect abundance to number of infected plants plus one, were formed by combining disease and abundance wave fronts (a). All curves were generated using Matlab (2018)

We begin by developing a simple qualitative theory of vector‐pulled, ‐pushed and ‐orthodox, landscape invasions of insect‐borne plant pathogens (Box 1, Figure 1). Central to the analysis is the idea of wave profiles and their associated patterns that are characteristic of vector‐pulled, ‐pushed and ‐orthodox, landscape invasions. We also outline a landscape simulation method, of insect colony and pathogen dynamics, for implementing three scenarios of insect superabundance: PMiS, EMiS and INViS. The landscape simulation is used to demonstrate that the signature of superabundance scenarios (cf. wave profiles) can be recovered from empirical data (Results section). We also outline a simple Bayesian approach to hypothesis testing for linking the theory to landscape datasets.

BOX 1 **‘Pulled’, ‘pushed’ and ‘orthodox’ invasions of insect‐borne plant pathogens**

In landscape invasions waves of plant disease are routinely linked to high insect vector abundance. Nevertheless, several distinct scenarios for the relation between insect abundance and plant disease waves can be identified—as depicted in Figure 1a —with consequences for landscape patterns.

**Vector‐pulled wave:** When a plant disease wave front is *pulled* by a wave of high insect abundance (blue wave spreads behind dotted black wave in Figure 1a, then the *wave profile*, defined as the ratio of abundance to infected plant incidence, peaks ahead of the disease front (Figure 1b).

**Vector‐pushed wave:** When a disease front is *pushed* by a wave of high insect abundance (blue wave spreads in front of dashed black wave in Figure 1a), then the wave profile dips in the wake of the plant disease wave front (Figure 1c).

**Vector‐orthodox wave:** When disease fronts simply spread through high background abundance (blue wave spreads through solid black background in Figure 1a), then the wave profiles neither dip nor peak in relation to the disease front (Figure 1d).

### 2.1 Biological invasions and wave profile patterns

Theory of invasion types for biological invasions has shown that there are two key types of population waves associated with invasions (Stokes, 1976): ‘pulled waves’ are driven by growth and dispersal processes at the leading edge of the invasion where densities are low (Lewis, 2016). ‘Pushed waves’ are driven by the growth and dispersal processes further back in the wave where densities are higher (Lewis, 2016). In the present work, we adapt the theory of invasion types to the situation of plant pathogens borne by a superabundant insect vector. In our terminology, a ‘vector‐pulled’ invasion represents a plant disease invasion that is driven by vector growth and dispersal in advance of the plant disease wave front. In contrast, a ‘vector‐pushed’ invasion is a plant disease invasion that is driven by vector growth and dispersal behind the plant disease wave front. It should be noted that both the original theory of invasion types (Lewis, 2016; Stokes, 1976) and our extension here are distinct from and bear no relation to ‘push–pull’ cropping systems developed for pest management (Cook et al., 2007).

We define wave profiles as the ratio of vector abundance to incidence of infected plants. The contrasting invasion types discussed above lead to different *wave profile* patterns. The patterns are a consequence of the relative position of disease and vector wave fronts (Box 1, Figure 1a). In ‘vector‐pulled’ invasions the wave profile peaks ahead of the disease front (Box 1, Figure 1b), because the vector wave front spreads in advance of the disease wave front. In ‘vector‐pushed’ invasions the wave profile dips behind the disease wave front (Box 1, Figure 1c), because the vector wave front follows in the wake of the disease wave front. In ‘vector‐orthodox’ invasions the wave profile increases monotonically across the landscape (Box 1, Figure 1d), because the disease wave front simply spreads through a high background abundance of the insect vector (due to environmental suitability). The patterns shown in Figure 1 are illustrative in order to introduce the relevant theory that underlies our methods. The following step is to show theoretically, using a method of landscape simulation that we now introduce, that the three scenarios of insect superabundance produce wave profiles recoverable as vector‐pulled, ‐pushed or ‐orthodox (as later discussed in the Results section).

### 2.2 Landscape simulation of pathogen emergence

For simplicity, we model the landscape symbolically as a sequence of *n* fields arranged along a single dimension with equal spacing between fields. Central to the landscape formulation is a model of insect abundance at the scale of average individual plants in fields. This allows for feedbacks between pathogen infection of plants and abundance on plants, due to, for example, PMiS, and hence to superabundance at the landscape scale. In this way, abundance per average healthy and per average infected plant (i.e. S plant colony, I plant colony), and in addition per average exposed plant (to account for the delay between plant infection and the onset of infectiousness; i.e. E plant colony) follows,**S plant colony, field #j**

(1) $$\text{Adults}\;\frac{{\mathrm{dA}}_{j}^{S}}{\mathrm{dt}}={\text{Maturing}}_{j}-{\text{Death}}_{j}-\text{Dispersal}\;{\text{change}}_{j},$$

(2) $$\text{Nymphs}\;\frac{{\mathrm{dN}}_{j}^{S}}{\mathrm{dt}}={\mathrm{aA}}_{j}^{S}\left(1-\frac{A_{j}^{S}}{K}\right)-{\text{Death}}_{j}-{\text{Maturing}}_{j},$$

**E plant colony, field #j**

(3) $$\text{Adults}\;\frac{{\mathrm{dA}}_{j}^{E}}{\mathrm{dt}}={\text{Maturing}}_{j}-{\text{Death}}_{j}-\text{Dispersal}\;{\text{change}}_{j},$$

(4) $$\text{Nymphs}\;\frac{{\mathrm{dN}}_{j}^{E}}{\mathrm{dt}}={\mathrm{aA}}_{j}^{E}\left(1-\frac{A_{j}^{E}}{K}\right)-{\text{Death}}_{j}-{\text{Maturing}}_{j},$$

**I plant colony, field #j**

(5) $$\text{Adults}\;\frac{{\mathrm{dA}}_{j}^{I}}{\mathrm{dt}}={\text{Maturing}}_{j}-{\text{Death}}_{j}-\text{Dispersal}\;{\text{change}}_{j},\;$$

(6) $$\text{Nymphs}\;\frac{{\mathrm{dN}}_{j}^{I}}{\mathrm{dt}}={\mathrm{aA}}_{j}^{I}\left(1-\frac{A_{j}^{I}}{\mathrm{\varepsilon K}}\right)-{\text{Death}}_{j}-{\text{Maturing}}_{j},$$

for fields j=1..n. In Equations 1–6 ε represents pathogen modification of the insect carrying capacity of infected plants (ε>1 indicates improvement of the host plant resource). Note that Donnelly and Gilligan (2020) showed that the epidemiological mechanism that is most plausible for PMiS encompasses a change to infected plant carrying capacity (ε in Equation 6 cf. no ε in Equations 2 and 4; Donnelly & Gilligan, 2020). In Equations 1–6, dispersal change_*j*_ refers to the net change in the adult abundance per average field j healthy, exposed or infected plant through dispersal. Dispersing adult insects can move between plants in field j and they can also move to plants within field j−1 or field j+1. Note that, in order to avoid boundary effects, movement can occur between plants in field 1 and plants in field *n*—that is, the landscape consists of a ring of fields with dispersal of adult insect vectors occurring within fields and between neighbouring fields. See Supporting Information S1 for details and full equations (Equations S1.1–S1.6).

The density of pathogen‐infected plants, that is, the pathogen dynamics, is next formulated for a given abundance of the vector. For the majority of insect‐borne plant pathogens, the overall transmission rate to plants is proportional to the number of infected vectors that are feeding on individual healthy plants. In this way the epidemic is described by equations for the number of pathogen‐infected and pathogen‐exposed plants and for the number of pathogen‐infected vectors at time t, that is, I_j(t)_, E_j(t)_ and Y_j(t)_,

(7) $$\text{Pathogen exposed plants},\text{field}\#j\;\frac{{\mathrm{dE}}_{j}}{\mathrm{dt}}={\text{inoculation}}_{j}-{\text{incubating}}_{j}-{\text{death}}_{j},$$

(8) $$\text{Pathogen infected plants},\text{field}\#j\;\frac{{\mathrm{dI}}_{j}}{\mathrm{dt}}={\text{incubating}}_{j}-\text{loss of}\;{\text{infection}}_{j},$$

(9) $$\text{Pathogen infected vectors},\text{field}\#j\;\frac{{\mathrm{dY}}_{j}}{\mathrm{dt}}={\text{acquisition}}_{j}-\text{loss of}\;{\text{infection}}_{j}.$$

See Supporting Information S1 for the full equations (Equations S1.10‐S1.12). In Equations (7), (8), (9) epidemics are limited by the rate at which infected plants lose infectiousness, through mortality or removal by growers (known as roguing). In the first instance, for simplicity, we assume dead plants are replaced with healthy plants so that the total population of plants remains constant—but replacement planting material may alternatively be pathogen exposed or pathogen infected, see following paragraph. In addition, the infectious period of the vector is limited by the rate that vectors cease being infectious (the sum of the constant rates that vectors lose the pathogen, and natural mortality). See Supporting Information S1 for associated parameters, all of which are listed and defined in Table S1.1.

Note that an additional mode of transmission that may be important in perennial crop epidemics is the introduction of infection when cuttings are used for host planting material. For simplicity, we incorporated this in our models separately in Supporting Information S2. We briefly discuss this extended model in the Results section.

### 2.3 Statistical analysis of wave profile patterns

In order to assess empirical wave profiles for the peaking or dipping patterns that are indicative of vector‐pushed or vector‐pulled invasions, we developed a hypothesis test based on quadratic regression. The empirical datasets that are most relevant for our purposes typically comprise measurements from fields along a transect orthogonal to the disease wave front. The hypothesis test involves the testing of wave profile response data (i.e. ratio of abundance to incidence of infected plants along a transect) against a null hypothesis that no extremum, that is, minimum or maximum, occurs over distance along the transect studied. In this way the null hypothesis corresponds to an assumption of EMiS. The null hypothesis is rejected if there is sufficient evidence of extremum occurrence along the given transect. This corresponds to a credible interval for the turning point within the interval $\left[0,1\right]$ (where transect distance is scaled to the interval $\left[0,1\right]$ prior to the analysis). Rejection of the null hypothesis supports an alternative hypothesis of PMiS if the credible interval for curvature is fully positive, or, alternatively, INViS if the credible interval for curvature is fully negative.

Credible intervals for turning point and curvature are estimated using polynomial regression. The regression model incorporates transect distance of fields (from the invaded end of the transect). In addition, if longitudinal multi‐year data are available, a random effect can be included to account for correlations across years in the quality of individual fields (i.e. a random effects model). The turning point is a compound parameter, and therefore a Bayesian regression, which can produce posterior distributions for compound parameters, is well suited (Plassmann & Khanna, 2007). See Supporting Information S3 for a full description.

## 3 RESULTS

In this section landscape simulation is applied to three possible scenarios of insect superabundance (INViS vs. PMiS vs. EMiS) using parameters sourced from published studies for CMGs (Colvin et al., 2006; Holt et al., 1997; Storey & Nichols, 1938) (Table S5.1). Simulation wave profiles, i.e., ratio of abundance to infected plant incidence as a function of transect distance, are found to correspond to those of vector‐pulled, ‐pushed and ‐orthodox expansions. Real‐world datasets for severe cassava disease are then analysed to determine if they correspond to vector‐pulled, ‐pushed or ‐orthodox expansions. The analysis allows investigation of the superabundance scenarios underlying cassava disease expansion in East Africa.

### 3.1 Invasive vector insect superabundance (INViS) gives rise to vector‐pulled landscape invasions

**FIGURE 2:**
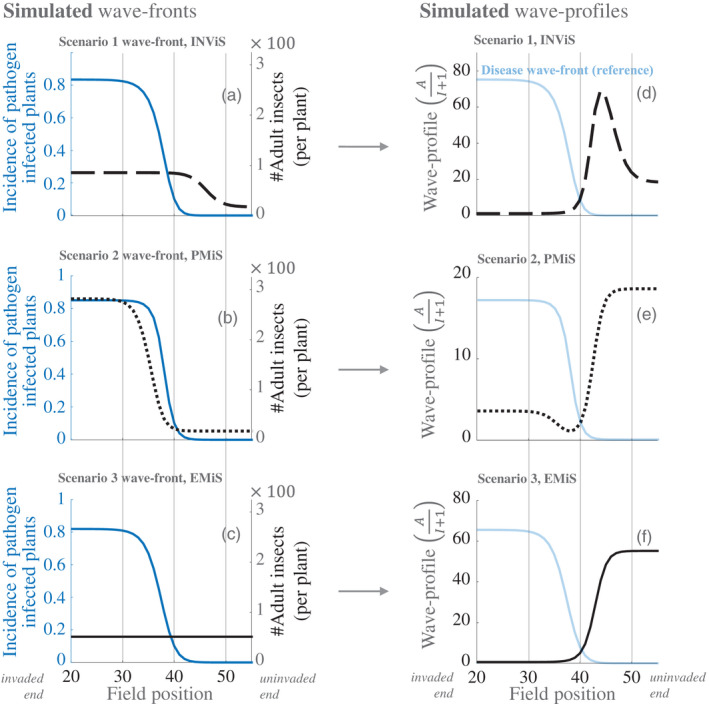
Pathogen emergence under three superabundance scenarios generate qualitatively similar disease fronts but contrasting wave profiles. Landscape simulation of vector and epidemiological dynamics when emergence is caused by: the arrival of a more fecund insect strain (a and d, INViS), by pathogen improvement of infected plant resource quality for vectors (b and e, PMiS), by enhanced environmental suitability for insect vectors across the landscape (c and f, EMiS). In a–c, black curves represent vector abundance within fields (right *y*‐axis) and blue curves represent the incidence of infected plants in a field (left *y*‐axis). In d–f, black curves represent wave profiles defined as the ratio of adult insect abundance to the number of infected plants within fields; light blue curves, for reference, represent disease fronts. The following rates, *per day*, were used to generate a–c: b=1/50, a=100b/2, $r^{\mathrm{acq}}=r^{\text{inoc}}=0.032$, θ=1, σ=0, μ=δ=1/360, $b_{N}=2b$, ν=1/30 and κ=1/25. The following probabilities were used: q=u=0.5 and H was 100 plants per field. Values for plant carrying capacity (K) and pathogen modification (*ε*) were chosen to produce comparable disease fronts: In a ε=1 and K=200 for an invading insect vector strain (K=40 for wild‐type vector); in b *ε* = 20 and K=40; in c *ε* =1 and K=120. Snapshots, which were taken under the same criterion that the 40th field had reached 0.1 incidence, show equivalent epidemic waves (Figure 2a vs. 2b vs. 2c). Note that insect abundance per plant at the uninvaded end of the landscape in Figure 2c is more than twice that of Figure 2a,b reflecting the landscape‐wide higher environmental suitability (×3 baseline carrying capacity). Simulations were run in MATLAB (2018)

To represent INViS, we conducted landscape simulations incorporating an invasive strain of the insect vector. Initially landscape dynamics were allowed to reach steady‐state levels of wild‐type vector abundance. From this initial condition, we seeded an individual insect vector representing an invasive strain associated with a carrying capacity on all host plants which is five times that of the wild‐type vector. We also seeded an individual plant infection with a pathogen having no effect on the vector carrying capacity of infected plants. Simulations lead to joint travelling waves across the landscape of plant disease and vector abundance (Figure 2a). Corresponding wave profiles feature a peak in advance of the disease wave front (Figure 2d)—as predicted for vector‐pulled invasions (cf. Box 1, Figure 1b).

### 3.2 Pathogen‐mediated insect superabundance (PMiS) gives rise to vector‐pushed landscape invasions

To represent PMiS, we conducted landscape simulations incorporating a mutant strain of the plant pathogen. Initially landscape dynamics were allowed to reach steady‐state levels of vector abundance. From this initial condition, we seeded an individual plant infection with a pathogen that increases the insect carrying capacity of infected plants by a multiplicative factor (ε=20). Simulations lead to joint travelling waves across the landscape of plant disease and vector abundance (Figure 2b). Corresponding wave profiles feature a dip in the wake of the disease front (Figure 2e), as predicted for vector‐pushed invasions (cf. Box 1, Figure 1c).

### 3.3 Environmentally mediated insect superabundance (EMiS) gives rise to vector‐orthodox, rather than vector‐pushed, or vector‐pulled, landscape invasions

To represent EMiS, we conducted landscape simulations incorporating high environmental suitability for insect growth (i.e. by simulating high plant carrying capacity for insect vectors across the landscape). Initially landscape dynamics were allowed to reach steady‐state levels of vector abundance for a landscape incorporating baseline environmental suitability. From this initial condition, we increased the carrying capacity of all host plants across the landscape by a factor of three to represent high environmental suitability. We also seeded an individual plant infection with a pathogen having no effect on the vector carrying capacity of infected plants. Simulations lead to a travelling wave of plant disease across the landscape while vector abundance is simultaneously high in all fields (Figure 2c). The corresponding wave profiles show a monotonic increase moving from invaded to uninvaded ends of the landscape (Figure 2f), that is, displaying neither an internal peak nor an internal dip, as predicted for vector‐orthodox invasions (cf. Box 1, Figure 1d). The wave profile patterns that are associated with PMiS, INViS and EMiS are summarised in Table 2, and the key terms are summarised in Table 3.

Our landscape model in the main text excludes transmission of the virus through plant cuttings for ease of presentation (and for applicability to a broader range of pathosystems). We analyse the impact of cutting transmission in the Supplementary Information where we distinguish two categories of cutting transmission. In the first category farmers are assumed to give priority to selecting asymptomatic planting material. In the second category farmers do not discriminate in selecting planting material. We also distinguish between locally sourced cuttings and landscape‐sourced cuttings. We find that the characteristic wave profile patterns for all scenarios are qualitatively unaffected by the inclusion of infection through plant cuttings, that is, the wave profile patterns from Figure 2 main text, as summarised in Table 2 main text, are preserved throughout Figures S2.1 and S2.2.

### 3.4 Two East African landscape datasets show a vector‐pushed pattern for invasion of severe cassava mosaic disease

**FIGURE 3:**
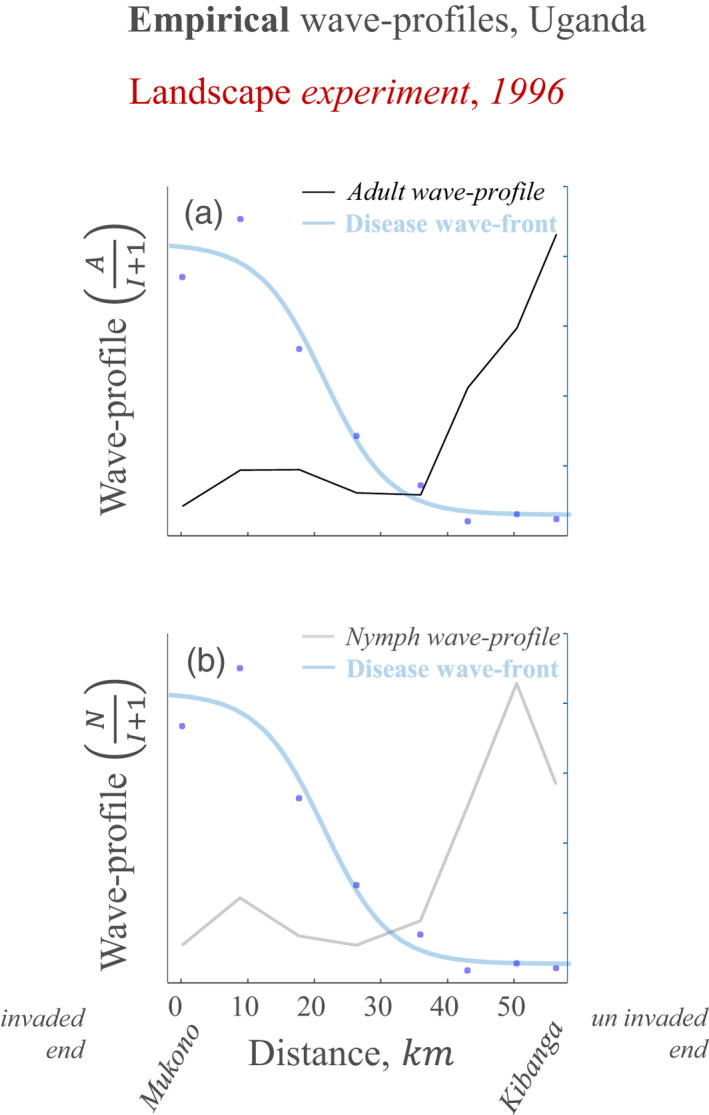
A whitefly‐borne geminivirus displays empirical wave profiles typical of vector‐pushed invasions along a landscape experiment invasion path. The empirical wave profiles are composed of data‐points taken from Colvin et al. (2004)’s experimental study of a regional severe cassava mosaic disease epidemic in 1996, the year prior to endemic severe disease occurring across the landscape transect. Black curve (a) represents the adult wave profile, defined as the ratio of adult insect abundance to the number of infected plants within fields; grey curve (b) represents the nymph wave profile, defined as the ratio of nymph insect abundance to the number of infected plants within fields; blue markers (a and b) represent the data‐points for incidence of infected plants. Blue curve (a and b), indicating disease front, was generated for reference using least squares fitting of a logistic curve through the data‐points

Analysis of experimental landscape data confirmed a dipping wave profile, indicating a vector‐pushed scenario (Figure 3). In 1996–1997, Colvin and co‐authors (Colvin et al., 2004) cultivated eight cassava plots along a 58 km Ugandan transect, orthogonal to an approaching cassava mosaic disease wave. We calculated wave profiles from the original published data, and found that the data display a ‘vector‐pushed’ pattern for 1996 (Figure 3a cf. Figure 2e), when the disease was emerging (Colvin et al., 2004). Although our main focus is on the wave profile formed from adult *B. tabaci*, a qualitatively similar wave profile was found for nymphs (Figure 3b; as is predicted in simulations, Figure S1.1).

In order to formalise the evidence for a vector‐pushed pattern, we applied the hypothesis testing approach (methods section) to the landscape experiment data (Colvin et al., 2004). This was designed to test for the occurrence of an internal minimum or maximum in the wave profile relative to the transect. Accordingly, the wave profile was found to have an internal minimum in 1996 (positive 95% credible interval for curvature, i.e. $\alpha_{2}\;95\%\;\mathrm{CI}>0$; 95% credible interval for turning point confined to transect, i.e. $0<\alpha^{\prime }\;95\%\;\mathrm{CI}<1$, see Table 1 epidemic 1996). No internal maximum/minimum, however, was found for 1997 (95% credible intervals for curvature and turning point both overlap with 0, see Table 1 endemic 1997). This is consistent with the observation made in Colvin et al. (2004) that the epidemic of severely infected plants had progressed such that the transect was considered within the pandemic area by 1997. Thus, hypothesis testing confirmed a dipping wave profile, indicative of a vector‐pushed scenario, when the disease was emerging along the landscape transect.

**TABLE 1:** Hypothesis testing of wave profile patterns for cassava mosaic disease. Statistical analyses test the null hypothesis that no wave profile extremum (i.e. minimum or maximum over transect distance) occurred in the transect studied. In A, likelihood ratio (LR) tests were performed to support a wave profile model that was quadratic in landscape distance, prior to performing extremum analyses (B). Statistics are reported, in A, for LR tests including *p*‐values (i.e. probability simpler model fits data as well as the more complex model), and, in B, for wave profile extremums comprising 95% credible intervals (CIs) for curvature ($\alpha_{2}\;95\%\;\mathrm{CI}$, B row 1) and turning point ($\alpha^{’}\;95\%\;\mathrm{CI}$, B row 2, where $\alpha^{\prime }=-\alpha_{2}/\alpha_{1}$). In A‐B statistically significant results, evaluated at the 95% confidence level, are highlighted in bold. Transect distance was first scaled to the interval [0,1] with the invaded side of the transect corresponding to 0 and the uninvaded end corresponding to 1. For the landscape experiment data (Colvin et al., 2004): Two years of repeated measures were available requiring a single mixed effects model (single landscape experiment entry, A), and, in addition, LR tests supported a common intercept across the 2 years. For the landscape survey data (Legg & Ogwal, 1998): The data spanned a single year and hence a fixed effects model for each survey transect was used (two landscape survey entries in A; see supporting information S4). LR tests on nested models were performed using the ‘lrtest’ command in R (package lmtest). Posterior parameter distributions for $\alpha_{2}$ and $\alpha^{\prime }$ were calculated using rStan (computer code can be accessed through link in code Availability section). Bayesian modelling was implemented in rStan v2.21.0. All analyses were carried out in R version 3.63 (R Core team, 2014)

		Landscape experiment		Landscape survey	
		Epidemic 1996	Endemic 1997	Epidemic central	Epidemic eastern
(A) Nonlinearity of wave profile					
Linear versus quadratic	$\chi^{2}$, *df*	11.766,2		5.58,1	9.545,1
	*p*‐value	**0.003**		**0.018**	**0.002**
Quadratic versus cubic	$\chi^{2}$, *df*	3.512,2		0.166,1	0.076,1
	*p*‐value	0.173		0.683	0.783
(B) Wave profile extremum					
Curvature	$\alpha_{2}$ 95% CI	[**0.419,1.561**]	$\left[-0.014,1.172\right]$	$\left[-0.024,0.139\right]$	[**0.003, 0.077**]
Turning point	$\alpha^{\prime }$ 95% CI	[**0.042, 0.4**]	$\left[-0.406,0.623\right]$	$\left[-2.079,2.168\right]$	[**0.163, 0.632**]

**FIGURE 4:**
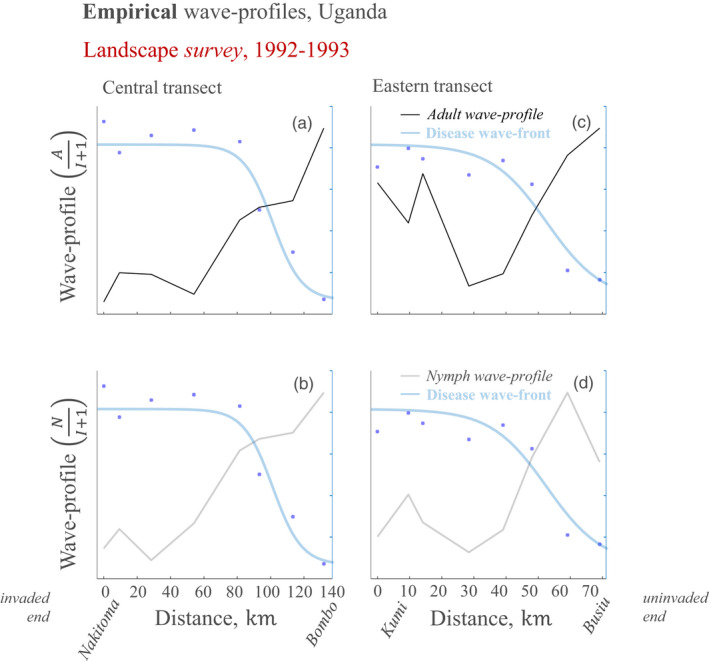
A whitefly‐borne geminivirus displays empirical wave profiles typical of vector‐pushed invasions along landscape survey invasion paths. The empirical wave profiles are composed of data‐points taken from Legg and Ogwal (1998)’s survey of a regional severe cassava mosaic disease epidemic in 1992–1993, averaged over the months in which the surveys were recorded. Black curves (a and c) represent adult wave profiles, defined as the ratio of adult insect abundance to the number of infected plants within fields; grey curves (b and d) represent nymph wave profiles, defined as the ratio of nymph insect abundance to the number of infected plants within fields; blue markers (a–d) represent the data‐points for incidence of infected plants. Blue curves (a–d), indicating disease front, were generated for reference using least squares fitting of a logistic curve through the data‐points

Analysis of a separate landscape survey dataset (Legg & Ogwal, 1998) showed similar patterns (Figure 4). In 1992–1993, Legg and Ogwal (1998) conducted a survey of a more northerly region of Uganda (relative to the later experimental study of Colvin et al. (2004)). The survey data, which were recorded for a selection of months between 1992 and 1993 spanning a period of approximately 1 year, were based on locally farmed cassava plants [in contrast to the identically planted fields of Colvin et al. (2004)]. In addition, survey data were collected for two, ‘eastern’ and ‘central’ transects (see Supporting Information S5 for a summary of Data Sources). Amendments to our testing procedure were necessary in order to take account of the following survey‐related factors: non‐longitudinal field observations and urban conurbation effects (see Supporting Information S4). In spite of the complexity inherent in the survey data, a ‘dipping’ pattern was also apparent for adult *B. tabaci* wave profiles for both central and eastern transects (Figure 4a–d). The patterns were confirmed by the statistical analysis for the eastern transect (positive 95% credible interval for curvature, i.e. $\alpha_{2}\;95\%\;\mathrm{CI}>0$; 95% credible interval for turning point confined to transect, i.e. $0<\alpha^{\prime }\;95\%\;\mathrm{CI}<1$, Table 1 eastern transect). The patterns were poorly supported, however, by the statistical analysis for the central transect (although the 95% credible intervals for turning point and curvature overlapped with zero, Table 1 central transect, it was found that a 88% credible interval supported a positive curvature). The poor support may be a consequence of greater spacing between locations for the central transect only (*c*. 20 km central transect cf. *c*. 10 km eastern transect and landscape experiment). The same overall patterns were found for survey wave profiles formed from *B. tabaci* nymph (Figure 4b,d).

Overall, the survey wave profiles closely resemble those of the landscape experiment (Figure 4 cf. Figure 3). The invaded end of the adult wave profile for the eastern transect, however, appears relatively high (Figure 4c) although this is not the case for the nymph wave profile (Figure 4d). The difference in adult and nymph wave profiles at these locations may indicate relatively high density of immigrating adult whitefly. In Legg and Ogwal (1998), the authors noted that clean planting material was deployed at the first two locations (Kumi and Atutur) on the eastern transect, while Otim‐Nape et al. (2001) report that the dominant variety in 1990–1992 was gradually replaced by a number of varieties. As such, alternative varieties having relative attractiveness for dispersing whitefly may be one possible explanation for the apparent high density of immigrating adult whitefly at Kumi and Atatur.

## 4 DISCUSSION

In order to investigate the landscape emergence of plant pathogens that are transmitted by superabundant insects, we studied three superabundance scenarios. The superabundance scenarios were: PMiS, INViS and EMiS. We analysed the superabundance scenarios using a theory of invasion types. The theory distinguishes plant disease waves that are pushed or pulled by a wave of superabundant insects, from orthodox disease invasions in which the background vector abundance is generally high. Epidemiological simulations demonstrated that each of the superabundance scenarios is characterised by different landscape profiles involving vector and pathogen densities. Accordingly, when we examined empirical data for cassava mosaic disease in field plots distributed along an invasion path, we found a wave profile pattern indicative of vector‐pushed invasions. A survey of farmer‐cultivated cassava along two transects exhibited a similar pattern.

In this paper, we used a landscape model to show that joint travelling waves of plant disease and high insect abundance spread across a simulated landscape if virus‐infected plants have a high insect vector carrying capacity compared with relatively low carrying capacity of uninfected plants (Figure 2b). For the same model system, epidemic spread does not occur if uninfected and virus‐infected plants have the same low carrying capacity, thus demonstrating that regional emergence of a plant virus can occur through PMiS. In addition, the PMiS, INViS and EMiS mechanisms produce indistinguishable epidemic curves depending on the relative size of carrying capacity alterations associated with each mechanism (Figure 2a–c). As such, all else being equal, epidemic spread may be more sensitive to changes in the carrying capacity of individual plants due to EMiS than INViS, and in turn more sensitive to changes due to INViS than PMiS. Early theoretical work on INViS and PMiS in Holt and Colvin (2001), analysing an equation for the production of infective emigrant whitefly, found that increased carrying capacity has more impact when it applies to all plant types rather than just infected plants, and this is consistent with our results (Figure 2a–c). Our main result then applied the landscape models to historical datasets: showing that the signature pattern of PMiS was evident in survey and experimental data dating from the emergence of severe cassava mosaic disease in Uganda.

### 4.1 High *B. tabaci* abundance and severe cassava mosaic disease

In the following paragraphs we briefly summarise several key initial findings from published studies relating to the INViS and PMiS hypotheses. We then describe how more recent studies have advanced these hypotheses—and finally we assess the evidence in light of our results.

Colvin et al. (2004) reported that the numbers of adults and nymphs of *B. tabaci* were positively correlated at each site in the same cassava mosaic disease‐based landscape experiment that is reanalysed here. They also made the observation that high *B. tabaci* populations were generated on severely diseased plants, and, as such, severely diseased plants were suitable for *B. tabaci* oviposition and development. This is an important observation, as the expectation that severely infected plants would be poor hosts for insect development has been an obstacle in the perceived feasibility of the PMiS hypothesis. In addition, the observation is supported here by the consistency of wave profile patterns for both nymphs and adult *B. tabaci* (Figures 3 and 4). In a subsequent paper Colvin et al. (2006) showed these densities are greater when breeding on symptomatic cassava leaves than on the leaves of healthy plants in particular for severe cassava mosaic disease (CMD; linked to co‐infection of ACMV and EACMV‐UG strains). As a whole, these studies therefore provide evidence that the high insect populations found in the field were associated with the severely diseased plants—which is consistent with PMiS. Based on the landscape survey herein reanalysed, Legg and Ogwal (1998) concluded that the wave front of severe cassava mosaic disease was likely a consequence of the large *B. tabaci* populations at the front—but that the explanation for the large populations themselves remained unclear. Using data collected in 1997 and 1999 from a similar study region to that of Colvin et al. (2004), Legg et al. (2002) found that a distinct *B. tabaci* genotype cluster, subsequently referred to as SSA2, was associated with the CMD epidemic—which is consistent with INViS.

SSA2 is one of five *B. tabaci* species found colonising cassava in sub‐Saharan Africa (SSA1 to SSA5; Ally et al., 2019). Although high *B. tabaci* abundance has persisted on cassava regionally (Ally et al., 2019), SSA2 has rarely been found in significant numbers in the region subsequent to the initial report by Legg et al. (2002) (Ally et al., 2019; Legg et al., 2014; Mugerwa, Colvin, et al., 2021; Mugerwa, Sseruwagi, et al., 2021). As such, published experimental evidence since the initial expansion has tended not to support INViS. Recent reports, however, of SSA2 on cassava in the drier and hotter regions of northern Uganda (Mugerwa, Colvin, et al., 2021; Mugerwa, Sseruwagi, et al., 2021) and nearby south Sudan (Misaka et al., 2020), has rendered it feasible that *B. tabaci* SSA2 moved southwards with the initial CMD expansion—but did not ultimately displace *B. tabaci* SSA1 species due to a lack of adaptation to the environment of central Uganda.

When we examined wave profiles from survey and experiment we did not find any evidence for a vector‐pulled wave (the absence of pre‐disease front peak, Figures 2 and 3; Table 1; Table 2). This represents a timely addition, based on published data from the original expansion, to the existing weight of evidence against INViS. By contrast, we did find evidence of a vector‐pushed wave in both the central region of Uganda cultivated in Colvin et al. (2004) and the relatively more northern region surveyed in Legg and Ogwal (1998; Figures 2 and 3a; Tables 1 and 2). This represents new evidence at the landscape scale for the role of PMiS in CMD disease expansion. In this work we analysed published datasets of historical importance in describing the initial emergence of severe CMD associated with EACMV‐Ug in East Africa, that is, the original expansion for which PMiS and INViS have been suggested. Analysis of datasets for subsequent phases of the expansion (see e.g. Szyniszewska et al., 2017) may provide a valuable point of comparison with the initial emergence of severe CMD in Uganda but are beyond the scope of this paper.

**TABLE 2:** Summary of characteristic patterns for wave profiles associated with vector‐pushed, vector‐pulled and vector‐orthodox landscape invasions. Wave profiles are defined as the ratio of insect abundance to the incidence of pathogen‐infected plants. The pattern of wave profiles (i.e. dips vs. peaks vs. monotonic increase), occurs on a transect orthogonal to the disease wave front and describes the change in the wave profile moving from invaded to uninvaded ends of the transect

Scenario	Wave profile	INViS	PMiS	EMiS
Vector‐pulled	Peaks ahead of disease front	✓	−	−
Vector‐pushed	Dips in wake of disease front	−	✓	−
Vector‐orthodox	Monotonic increase	−	−	✓

**TABLE 3:** Glossary of key terms used in this study

INViS, Invasive vector insect superabundance
Invasive insect growth rate higher than for wild‐type insect vector, leading to superabundance
PMiS, Pathogen‐mediated insect Superabundance
Insect growth rate elevated on pathogen‐infected plants, leading to insect superabundance
EMiS, Environment‐mediated insect Superabundance
Insect growth rate elevated on all plants due to high environmental suitability
CMG, Cassava Mosaic Geminivirus
Multiple species of plant virus in the genus *Geminivirus*; vectored by *Bemisia tabaci* whitefly

### 4.2 High *B. tabaci* abundance and environmental factors

An additional study using CLIMEX modelling provides a picture of the ecoclimatic situation in which the CMD epidemic has been unfolding. Focusing on the East African region, Kriticos et al. (2020) demonstrated a clear correlation between cassava mosaic disease proliferation and a 39 year change in environmental suitability for a *B. tabaci* whitefly species. The species in question, Middle East‐Asia Minor 1, has not been recorded as a pest of cassava (Kriticos et al., 2020). Assuming *B. tabaci* species SSA1–SSA5 are subject to similar environmental trends, we separate out several ways in which invasion of severe CMD may have been influenced by the changing environment. Increasing environmental suitability may lead to *B. tabaci* invasion in a previously uncolonised region (route 1), or to an increase in colony size in an already colonised region (route 2). Alternatively, an increase in environmental suitability may enlarge the domain over which disease invasion occurs, that is, the domain of expansion is merely constrained by environmental suitability (route 3).

How do the three routes relate to our results? The first route, essentially describing a vector‐pulled wave, corresponds to an INViS scenario. When we examined wave profiles from survey and experiment we did not find any evidence for a vector‐pulled wave. The second route corresponds to an EMiS scenario—that is, is independent of a high‐growth invasive strain of the insect vector (vector‐pulled wave; INViS), or, pathogenic improvement of infected host plants as a resource for insect vector growth (vector‐pushed wave; PMiS). It involves the gradual increase in suitability over time leading to a gradient in abundance. We cannot rule the second route out (the EMiS null hypothesis was not rejected in the central survey transect, Table 1), however, on balance, the second route appears not to be supported by our results, and also appears contrary to historical reports of a *B. tabaci* wave spreading across the region (Otim‐Nape & Thresh, 1998).

The third route, that is, disease expansion occurring through dynamic processes in a domain that is constrained by environmental suitability, is consistent with the wave profile analysis in this paper. As such, the pattern of the wave profiles provides new evidence suggesting a role for PMiS in the dynamics of the expansion itself, while it is likely that the domain in which the expansion has been occurring has been determined by a changing environment. It is important to note, however, that subsequent persistence of high *B. tabaci* abundance may involve a range of factors (Macfadyen et al., 2021), including climate dynamics (Kriticos et al., 2020), land use (Kalyebi et al., 2021), and, in particular, the deployment of CMD‐tolerant cassava varieties, which are now known to be highly susceptible to *B. tabaci* (Katono et al., 2021).

## 5 CONCLUSIONS

We demonstrated a common pattern in the wave profiles of a landscape experiment and a landscape survey. The pattern constitutes evidence that PMiS was a dynamic driver in the East African expansion of severe cassava mosaic disease—although note that the null hypothesis (representing EMiS) was not rejected for one survey transect where the spatial resolution was relatively weak. The results presented in Kriticos et al. (2020) suggest that the expansion has occurred against a background of regional climate change.

Our analyses have adapted a well‐established theory of invasion types that is known for its implications for invasion wave‐speed (Stokes, 1976). For instance, the wave‐speeds of pushed and pulled expansions differ in that only wave‐speeds of pushed expansions are influenced by the invading organism’s carrying capacity (Gandhi et al., 2016; Lewis, 2016). Therefore, in light of our conclusions here, investigation of the factors controlling the wave‐speed of vector‐pushed disease invasions may lead to entirely novel insights—that may be highly relevant to the control of cassava mosaic disease expansion at continental scales.

## CONFLICT OF INTEREST

The authors declare no conflict of interest.

## AUTHOR CONTRIBUTIONS

R.D. and C.A.G. designed the study; R.D. performed the research and developed the methods. R.D. and C.A.G. wrote the manuscript.

## Supporting information


Supinfo1

Supinfo2

Supinfo3

Supinfo4

Supinfo5

## Data Availability

Supporting computer code and data is available on GitHub through Zenodo https://zenodo.org/record/5949262#.Yfv8hurP3IU Donnelly & Gilligan, 2022).
