# Supplementary material for: The role of pathogen‐mediated insect superabundance in the East African emergence of a plant virus
Source: J Ecol. 2022 Mar 13;110(5):1113–24. doi: 10.1111/1365-2745.13854 (PMC9310957; doi:10.1111/1365-2745.13854)
Supplement: Supplementary file 1 — Supinfo1 [file JEC-110-1113-s003.pdf]

Donnelly and Gilligan The role of pathogen mediated insect superabundance in the east-African emergence of a plant virus

Supporting information contains mathematical derivations for the landscape simulation model (Supporting Information S1 for main model and Supporting Information S2 for model incorporating pathogen-infected host plant cuttings), details of the hypothesis testing approach developed to analyse wave-profiles (Supporting Information S3), details of methods used to analyse survey data (Supporting Information S4) and details of data sources (Supporting Information S5).

Supporting Information S1, Pathosystem dynamics

The modelling framework of Donnelly and Gilligan [2], which links the population dynamics of insect vector colonies on individual plants to virus acquisition and inoculation during feeding, is extended here to a series of fields within a landscape linked through vector dispersal. The ensuing landscape framework was used to simulate the three superabundance scenarios (main text). We here derive equations for the pathosystem dynamics.

Colony dynamics

In order to investigate the landscape invasion of plant pathogens in relation to insect superabundance we model the joint population dynamics of insect colonies and pathogen epidemics. Phytophagous insect vectors of plant pathogens like whitefly, aphids and thrips, move between host plants in order to settle and feed on plant veins via the phloem and, when settled, to reproduce. To encompass pathogen mediated insect superabundance (PMiS), we incorporate pathogen modification of plants, which affects the population dynamics of vectors, leading to relatively high vector abundance on infected compared with

healthy plants. Fundamentally, the insect population dynamics involve reproduction, mortality and dispersal with density dependence constraining population growth of the vector at the level of individual plants [1, 2]. We considered a sequence of w fixed populations of H plants representing individual fields situated along a symbolic 1-dimensional, ring-shaped, landscape comprising healthy ($S_j(t)$), pathogen-exposed ($E_j(t)$), and pathogen-infected ($I_j(t)$) individual plants (i.e., $S_j(t) = H - E_j(t) - I_j(t)$) within individual fields (indexed by j). Generalising the framework of Donnelly and Gilligan [2] (for dynamics within fields) to the situation of dynamics within and between a sequence of fields, leads to the following equations for A_j^S, A_j^E and A_j^I (adult vector density on the average healthy, exposed and infected plant, respectively, in field j) and for N_j^S, N_j^E and N_j^I (nymph vector density on the average healthy, exposed and infected plant, respectively, in field j):

S plant colony, field #j

$$\textbf{Adults} \quad \frac{dA_j^S}{dt} = \overbrace{\kappa N_j^S}^{\text{Development}} - \overbrace{bA_j^S}^{\text{Death}} - \overbrace{\theta A_j^S}^{\text{Dispersal}} + \overbrace{q\theta(A_j^S S_j + A_j^E E_j + A_j^I I_j)\frac{1}{H}}^{\text{Dispersal}} + \overbrace{\theta\frac{(1-u)(1-q)}{2}\psi_j\frac{1}{H}}^{\text{Between field dispersal}}, \tag{S1.1}$$

$$\textbf{Nymphs} \quad \frac{dN_j^S}{dt} = \underbrace{aA_j^S(1 - \frac{A_j^S}{\epsilon_S K})}_{\text{Reproduction}} - \underbrace{b_N N_j^S}_{\textit{Death}} - \underbrace{\kappa N_j^S}_{\textit{Development}}, \tag{S1.2}$$

E plant colony, field #j

Adults
$$\frac{dA_j^E}{dt} = \kappa N_j^E - bA_j^E - \theta A_j^E + q\theta(A_j^S S_j + A_j^E E_j + A_j^I I_j)\frac{1}{H} + \theta\frac{(1-u)(1-q)}{2}\psi_j\frac{1}{H}, \tag{S1.3}$$

Nymphs
$$\frac{dN_j^E}{dt} = aA_j^E(1 - \frac{A_j^E}{\epsilon_E K}) - b_N N_j^E - \kappa N_j^E, \tag{S1.4}$$

I plant colony, field #j

Adults
$$\frac{dA_j^I}{dt} = \kappa N_j^I - bA_j^I - \theta A_j^I + q\theta(A_j^S S_j + A_j^E E_j + A_j^I I_j)\frac{1}{H} + \theta\frac{(1-u)(1-q)}{2}\psi_j\frac{1}{H}, \tag{S1.5}$$

Nymphs
$$\frac{dN_j^I}{dt} = aA_j^I(1 - \frac{A_j^I}{\epsilon_I K}) - b_N N_j^I - \kappa N_j^I, \tag{S1.6}$$

for $j = 1, 2...w$. In equations S1.1-S1.6 a and K denote low-density net reproduction rate and the maximum vector density per plant for vector multiplication to occur, b denotes the natural mortality rate of vectors, θ denotes the rate of vector dispersal between plants, and κ denotes the maturation rate of nymphs. In addition, q is the probability that vectors disperse to alternative plants in field j as opposed to migrating outside the field, and u is the probability that vector migrations out of the field are lost from the ring of fields. Finally, $\epsilon_I = \epsilon > 1$ together with $\epsilon_S = \epsilon_E = 1$ accounts for increase of the resource quality of infected host plants for vectors if $\epsilon > 1$, by increasing plant carrying capacity for vectors, and ψ_j denotes the total number of dispersing insects from neighbouring fields. For simplicity, between-field dispersal occurs only between nearest neighbour fields (with

equal probability). Since the symbolic landscape is arranged as a ring:

$$\psi_j = \begin{cases} (A^S_{j-1}S_{j-1} + A^E_{j-1}E_{j-1} + A^I_{j-1}I_{j-1}) + (A^S_{j+1}S_{j+1} + A^E_{j+1}E_{j+1} + A^I_{j+1}I_{j+1}), & j = 2..w-1 \\ (A^S_w S_w + A^E_w E_w + A^I_w I_w) + (A^S_2 S_2 + A^E_2 E_2 + A^I_2 I_2), & j = 1 \\ (A^S_{w-1}S_{w-1} + A^E_{w-1}E_{w-1} + A^I_{w-1}I_{w-1}) + (A^S_1 S_1 + A^E_1 E_1 + A^I_1 I_1), & j = w \end{cases} . \tag{S1.7}$$

To encompass environment mediated insect superabundance (EMiS), we incorporate an elevated plant carrying capacity for the insect vector across the landscape (Eq.s 1.1-1.7 with $\epsilon_S = \epsilon_E = \epsilon_I = \epsilon > 1$). This is also the case for the invasive insect vector in a scenario of invasive vector insect superabundance (INViS) while the plant carrying capacity for the wild-type insect vector is not elevated.

Epidemiological dynamics

For the majority of insect-borne plant pathogens, the overall transmission rate to plants is proportional to the number of infected adult vectors that are feeding on healthy plants denoted $A_j^{S^+}$, i.e.,

$$\textit{inoculation rate, field j:} \qquad r^{inoc} S_j A_j^{S^+}, \tag{S1.8}$$

where, r^{inoc} is the per infected vector rate at which plants are inoculated during feeding. Conversely, the transmission rate to vectors is proportional to the number of uninfected adult vectors that are feeding on infected plants denoted $A_j^{I^-}$, i.e.,

$$\textit{acquisition rate, field j:} \qquad r^{acq} A_j^{I^-} I_j = r^{acq} I_j (A_j^I - A_j^{I^+}), \tag{S1.9}$$

where r^{acq} denotes the per uninfected vector rate at which the pathogen is acquired during feeding on infected host plants. Combining these terms, the landscape epidemic is described by equations for the number of pathogen-exposed and pathogen-infected plants and for the number of pathogen-infected vectors at time t in field j, i.e., $E_j(t)$, $I_j(t)$ as well as A_j^{S+}, A_j^{E+} and A_j^{I+} (denoting the number of infected adult vectors that are feeding on healthy, exposed and infected plants respectively, see Table S1.1 for a summary of parameters),

Pathogen exposed plants, field #j

$$\frac{dE_j}{dt} = \overbrace{r^{inoc} S_j A_j^{S+}}^{\text{Inoculation}} - \overbrace{\nu E_j}^{\text{Incubation}} - \overbrace{\mu E_j}^{\text{Death}} \tag{S1.10}$$

Pathogen infected plants, field #j

$$\frac{dI_j}{dt} = \overbrace{\nu E_j}^{\text{Incubation}} - \overbrace{(\mu + \delta) I_j}^{\text{Removal}} \tag{S1.11}$$

Pathogen infected vectors, field #j

$$\begin{aligned}
\frac{dA_j^{S+}}{dt} &= -\theta A_j^{S+} + \theta \frac{qY_j}{H} + \theta \frac{(1-u)(1-q)}{2} \gamma_j \frac{1}{H} - (\sigma + b) A_j^{S+} \\
\frac{dA_j^{E+}}{dt} &= -\theta A_j^{E+} + \theta \frac{qY_j}{H} + \theta \frac{(1-u)(1-q)}{2} \gamma_j \frac{1}{H} - (\sigma + b) A_j^{E+} \\
\frac{dA_j^{I+}}{dt} &= \underbrace{r^{acq} I_j (A_j^{I} - A_j^{I+})}_{\text{Acquisition}} - \underbrace{\theta A_j^{I+}}_{\text{Dispersal}} + \underbrace{\theta \frac{qY_j}{H}}_{\text{Dispersal}} + \underbrace{\theta \frac{(1-u)(1-q)}{2} \gamma_j \frac{1}{H}}_{\text{Immigration}} - \underbrace{(\sigma + b) A_j^{I+}}_{\text{Virus loss}},
\end{aligned} \tag{S1.12}$$

for field $j = 1, 2..w$, where $Y_j = A_j^{S+} S_j + A_j^{E+} E_j + A_j^{I+} I_j$ and where epidemics are limited by the rate at which infected plants cease being infectious, denoted $\mu + \delta$, through mortality

(μ) or removal by growers (δ known as roguing). We assume dead plants are replaced with healthy plants so that the total population of plants remains constant. In addition, the infectious period of the vector is limited by the rate that vectors cease being infectious (the sum of the constant rates that vectors lose the pathogen, σ, and natural mortality, b) and all parameters are listed and defined in Table $S1.1$. Finally, the total number of immigrating infected adult insects into field j, on a 1-dimensional ring landscape of w fields, is,

$$\gamma_j = \begin{cases} Y_{j-1} + Y_{j+1}, & \text{when } j = 2..w-1 \\ Y_w + Y_2, & \text{when } j = 1 \\ Y_{w-1} + Y_1, & \text{when } j = w \end{cases} \tag{S1.13}$$

Insect superabundance

The pathogen is carried between host plants by insect vectors as they disperse over landscapes. When we consider *PMiS*, we are referring to elevated insect abundance at the landscape scale that is associated with the incidence of pathogen-infected plants. Accordingly, the degree of pathogen-mediated insect superabundance, denoted $V(I)$, is defined in terms of the total population size of the vector in the population of host plants, as the conditional ratio,

Degree of PMiS

$$V_j(I_j) = \frac{(A_j^S(I_j) + N_j^S(I_j))S_j(t) + (A_j^E(I_j) + N_j^E(I_j))E_j(t) + (A_j^I(I_j) + N_j^I(I_j))I_j(t)}{(A_j^S(0) + N_j^S(0))H_j} \begin{cases} > 1 \text{ pathogen-mediated superabundance} \\ = 1 \text{ no effect of pathogen on abundance} \\ < 1 \text{ pathogen-mediated subabundance} \end{cases}, \quad \text{(S1.14)}$$

where H_j represents the total host plant population size in field j [2]. The magnitude (degree) of PMiS is high when vector population size in the endemic landscape, i.e. the numerator in Eq. S1.14, is high, compared with its size in the infection-free landscape, i.e., the denominator in Eq. S1.14.

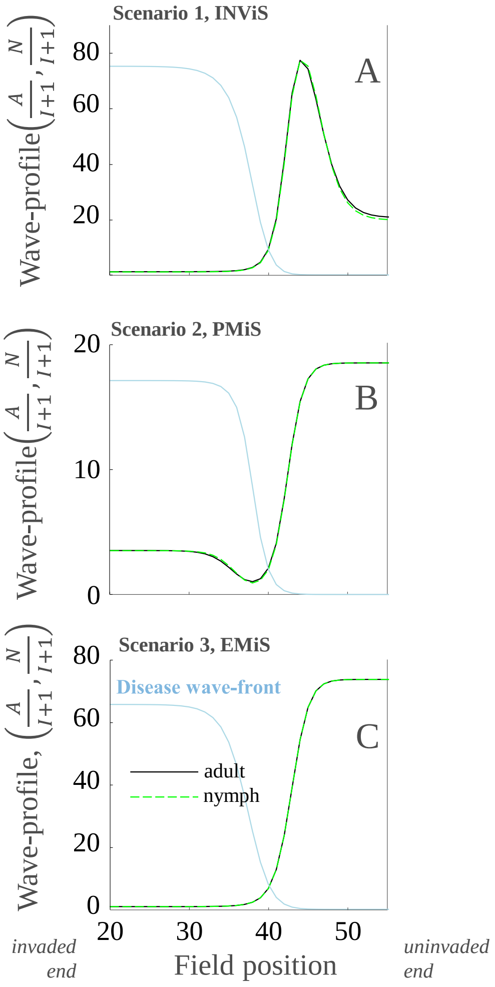

Figure S1.1: Pathogen emergence under three scenarios are associated with qualitatively distinct wave-profiles of both adult *and nymphal* insect vectors. Landscape simulations correspond exactly to those of Fig. 2. In A-C, black curves represent adult wave-profiles defined as the ratio of adult insect abundance to the number of infected plants within fields; green dashed curves represent nymphal wave-profiles defined as the ratio of nymph insect abundance to the number of infected plants within fields; blue curves, for reference, represent disease fronts. Note that to ensure the same scale nymph wave-profiles were normalised and scaled so that the maximum nymph wave-profile equals the maximum adult wave-profile.

**Definition of notation used** *(i) Population dynamics (plants, vectors)*		*Units*
Y_j	Number of infected adult vectors	*in field j*
S_j	Number of healthy plants	*in field j*
E_j	Number of exposed plants	*in field j*
I_j	Number of infected plants	*in field j*
A_j^i	Adult abundance per *average* i*-type* plant	*per plant field j*
N_j^i	Nymph abundance per *average* i*-type* plant	*per plant field j*
A_j	Total adult vector abundance	*in field j*
A_j^{i+}	Infected adult abundance per *average* i*-type* plant	*per plant field j*
A_j^{i-}	Uninfected adult abundance per *average* i*-type* plant	*per plant field j*
ψ_j	Total neighboring fields dispersing adults	*per field j*
γ_j	Total neighboring fields dispersing infected adults	*per field j*
(ii) Additional parameters		
ϵ	Modification of carrying capacity	*scaling factor*
μ	Healthy plant mortality rate	*per day*
δ	Rate of additional removal of infected plants	*per day*
ν	Incubation rate of infection in plants	*per day*
κ	Nymph vector development rate	*per day*
b, b_N	Adult, Nymph, vector mortality rate	*per day*
θ	Adult dispersal rate	*per day*
a	Adult reproduction rate (for 0 vector abundance)	*per day per capita*
K	Adult reproduction limit (density upper limit)	*max vector per plant*
r^{acq}	Rate of acquisition of pathogen	*per day*
r^{inoc}	Rate of inoculation of pathogen	*per day*
q	Probability vector dispersal remains in field	*probability*
u	Probability vector migration from field is lost	*probability*

Table S1.1: Summary of population variables and parameters. (i) The mathematical model tracks changes in plant and vector population variables. Pathogen mediated insect superabundance may arise through altered vector multiplication on infected plants (ϵ). (ii) Pathosystems are characterised by vector and pathogen life history parameters.

REFERENCES

1. Donnelly R, Cunniffe NJ, Carr JP, Gilligan CA. 2019 Pathogenic modification of plants enhances long-distance dispersal of nonpersistently transmitted viruses to new hosts. *Ecology*, 100:7, e02725.

2. Donnelly R, Gilligan CA. 2020. What is pathogen-mediated insect superabundance? *Journal of the Royal Society Interface*, 17(170), p.20200229.
