# Supplementary material for: The role of pathogen‐mediated insect superabundance in the East African emergence of a plant virus
Source: J Ecol. 2022 Mar 13;110(5):1113–24. doi: 10.1111/1365-2745.13854 (PMC9310957; doi:10.1111/1365-2745.13854)
Supplement: Supplementary file 2 — Supinfo2 [file JEC-110-1113-s002.pdf]

Supporting Information S2, Pathosystem dynamics, infection in plant cuttings

The landscape modelling framework is extended here to incorporate the possible spread of infection in host plant cuttings that are used to propagate the host crop. The colony dynamics are as per Supporting Information S1. The epidemiological dynamics are as per Supporting Information S1 but with additional rates of increase of pathogen-infected plants and/or pathogen exposed plants depending on the level of discrimination exercised by farmers in the selection of plant cuttings.

Epidemiological dynamics

The extended equations for epidemiological dynamics can take two forms. In the first form, farmers avoid propagating plant cuttings from infected plants. However, plant cuttings may yet originate in pathogen-exposed (though as yet not infectious) plants, leading to,

$$\begin{aligned}
\frac{dE_j}{dt} &= \overbrace{r^{inoc} S_j A_j^{S+}}^{\text{Inoculation}} - \overbrace{\nu E_j}^{\text{Incubation}} - \overbrace{\mu E_j}^{\text{Death}} \\
&\quad + \overbrace{(1-pC)\left((1-cW)\frac{E_j}{H-I_j} + cW\frac{\bar{E}}{H-\bar{I}}\right)(\delta I_j + \mu H_j)}^{\text{Growers are discriminating}} \\
\frac{dI_j}{dt} &= \overbrace{\nu E_j}^{\text{Incubation}} - \overbrace{(\mu+\delta) I_j}^{\text{Removal}}
\end{aligned} \tag{S2.1}$$

which we refer to as the *model with discriminating growers*. In the system of equations S2.1 the additional parameters pC and cW represent the proportion of confirmed clean seed used as cuttings, and the proportion of cuttings that are selected from the focal field vs the wider landscape respectively. In addition, $\bar{E}$ is the average incidence of pathogen-exposed plants over the wider landscape. Alternatively, farmers may pay little attention to the presence of pathogen infection in plant cuttings leading to,

$$
\begin{aligned}
\frac{dE_j}{dt} &= \overbrace{r^{inoc} S_j A_j^{S+}}^{\text{Inoculation}} - \overbrace{\nu E_j}^{\text{Incubation}} - \overbrace{\mu E_j}^{\text{Death}} \\
&\quad + \overbrace{(1 - pC)\left((1 - cW)E_j + cW\bar{E}\right)(\delta I_j + \mu H_j)}^{\text{Growers are not discriminating}} \\
\frac{dI_j}{dt} &= \overbrace{\nu E_j}^{\text{Incubation}} - \overbrace{(\mu + \delta) I_j}^{\text{Removal}} \\
&\quad + \overbrace{(1 - pC)\left((1 - cW)I_j + cW\bar{I}\right)(\delta I_j + \mu H_j)}^{\text{Growers are not discriminating}}
\end{aligned}
\tag{S2.2}
$$

which we refer to as the *model with growers who are not discriminating*, in which both pathogen-infected infected material and pathogen-exposed material may be planted. In the system of equations S2.2, in addition to the parameters that were introduced for the *model with discriminating growers*, $\bar{I}$ represents the average incidence of pathogen-infected plants over the wider landscape.

Selection of material for cuttings

In addition to discriminating vs not discriminating cultivation of cuttings, the cuttings in question may originate in the field in which they are planted ($cW = 1$), or they may

originate from planting material found across the landscape ($cW = 0$). Consideration of these two aspects, lead to four cases representing extreme scenarios for which we generate simulated wave-profiles (Fig S2.1-Fig S2.2 cf. Fig. 2, main text). The four cases are as follows:

- Case 1, *Growers are discriminating in the use of cuttings that originate from their field* (Fig S2.1 A-C)
- Case 2, *Growers are discriminating in the use of cuttings that originate from the wider landscape* (Fig S2.1 D-F)
- Case 3, *Growers are not discriminating in the use of cuttings that originate from their field* (Fig S2.2 A-C)
- Case 4, *Growers are not discriminating in the use of cuttings that originate from the wider landscape* (Fig S2.2 D-F)

Results

Our landscape model in the main text excludes transmission of the virus through plant cuttings for ease of presentation (and for applicability to a broader range of pathosystems). We analyse the impact of cutting transmission in this Supplementary Information where we distinguish two categories of cutting transmission. In the first category farmers are assumed to give priority to selecting asymptomatic planting material such that the transmission of CMV is proportional to the incidence of pathogen-exposed plants among healthy and pathogen-exposed plants (i.e., pathogen-infected plants are rejected; model corresponding to Eq. S2.1). In the second category farmers do not discriminate in selecting planting material so that transmission of the pathogen is proportional to the incidences of pathogen-infected and pathogen-exposed plants (model corresponding to Eq.

S2.2). Within each of these categories we also distinguish between locally-sourced cuttings, assumed to originate in the same field that they are planted, and landscape-sourced cuttings, assumed to originate from an aggregate of pathogen incidence in fields across the landscape.

The above classification leads to four cases: Category 1 (farmers are discriminating in selecting planting material) includes case 1 (locally sourced cuttings, Eq. S2.1 with cW=1) and case 2 (landscape sourced cuttings, Eq. S2.1 with cW=0). Category 2 (farmers do not discriminate in selecting planting material) includes case 3 (locally sourced cuttings, Eq. S2.2 with cW=1) and case 4 (landscape sourced cuttings, Eq. S2.2 with cW=0). Application of the same analysis that is described in the main text for the theoretical model (see Methods section) to cases 1-2 and 3-4 resulted in Fig.S2.1 and Fig.S2.2, respectively. Note that the results from the main text (no transmission of the virus through plant cuttings) are reproduced as the left panel in Fig.S2.1- Fig.S2.2, with horizontal rulers, for ease of comparison.

In comparison with the case of no transmission of the virus through plant cuttings (Fig. S2.1-S2.2 A,D,G) we see that the asymptomatic incidence of infected plants is significantly higher when there is cutting transmission if farmers are not discriminating in the use of plant cuttings (case 3-4, Fig. S2.2 B,C and E,F and H,I cf. Fig S2.2 A and D and G respectively). When farmers are instead discriminating in the use of plant cuttings, then asymptotic incidence is only marginally higher than when there is no transmission of the virus through plant cuttings (case 1-2, Fig. S2.1 B,C and E,F and H,I cf. Fig S2.1 A and D and G respectively). The characteristic wave-profile patterns for all scenarios are qualitatively unaffected by the inclusion of infection through plant cuttings (the wave-profile patterns from Fig. 2 main text, as summarised in Table 2 main text, are preserved throughout Fig. S2.1-S2.2).

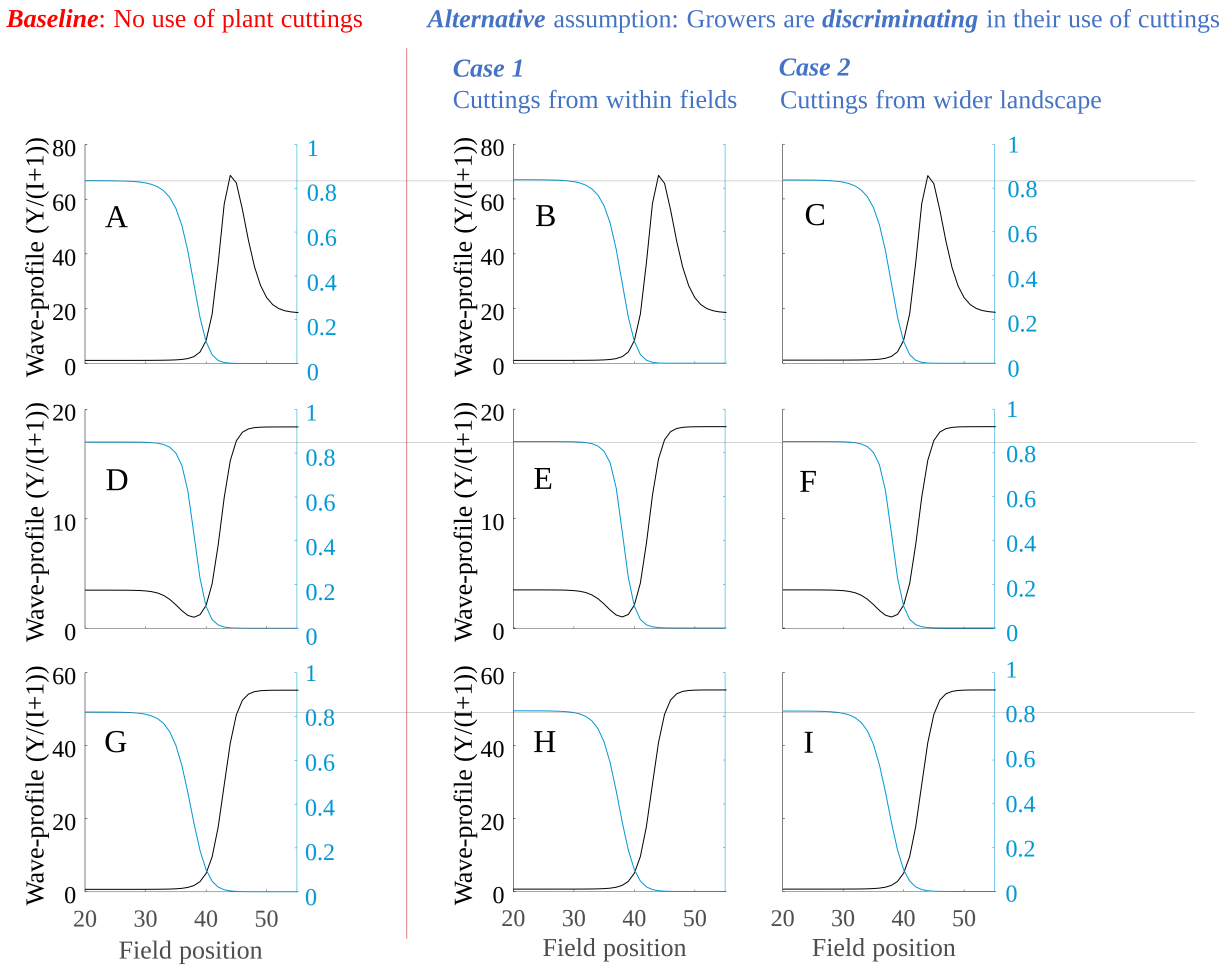

Figure S2.1: Pathogen emergence under three superabundance scenarios generate contrasting wave-profiles as identified in the main text (Fig. 2, Table 2) when propagation of the host crop involves potentially pathogen-exposed cuttings, i.e., where growers are discriminating in their use of cuttings. Landscape simulation of insect vector and epidemiological dynamics when emergence is caused by: the arrival of a more fecund insect strain (A-C, INViS), by pathogen improvement of infected plant resource quality for insect vectors (D-F, PMiS), by enhanced environmental suitability for insect vectors across the landscape (G-I, EMiS). In A, D and G the results from Fig. 2 main text which correspond to no infected cuttings are reproduced for comparison (equivalent to 100% clean seed, $pC = 1$ in Eq.s S2.1). In B-C, E-F and H-I where $pC = 0$, farmers are discriminating in their use of infectious cuttings; in B, E and H the cuttings solely originate from the grower's field (case 1, system S2.1 with $cW = 1$); in C, F and I the cuttings originate from the wider landscape (case 2, system S2.1 with $cW = 0$). In A-F, black curves represent wave-profiles defined as the ratio of insect abundance to the number of infected plants within fields; light blue curves, for reference, represent disease fronts. The parameters were otherwise as described in caption of Fig. 2, main text. Snapshots were taken when the 40th field had reached 0.1 incidence. Simulations were run in MATLAB R2018a.

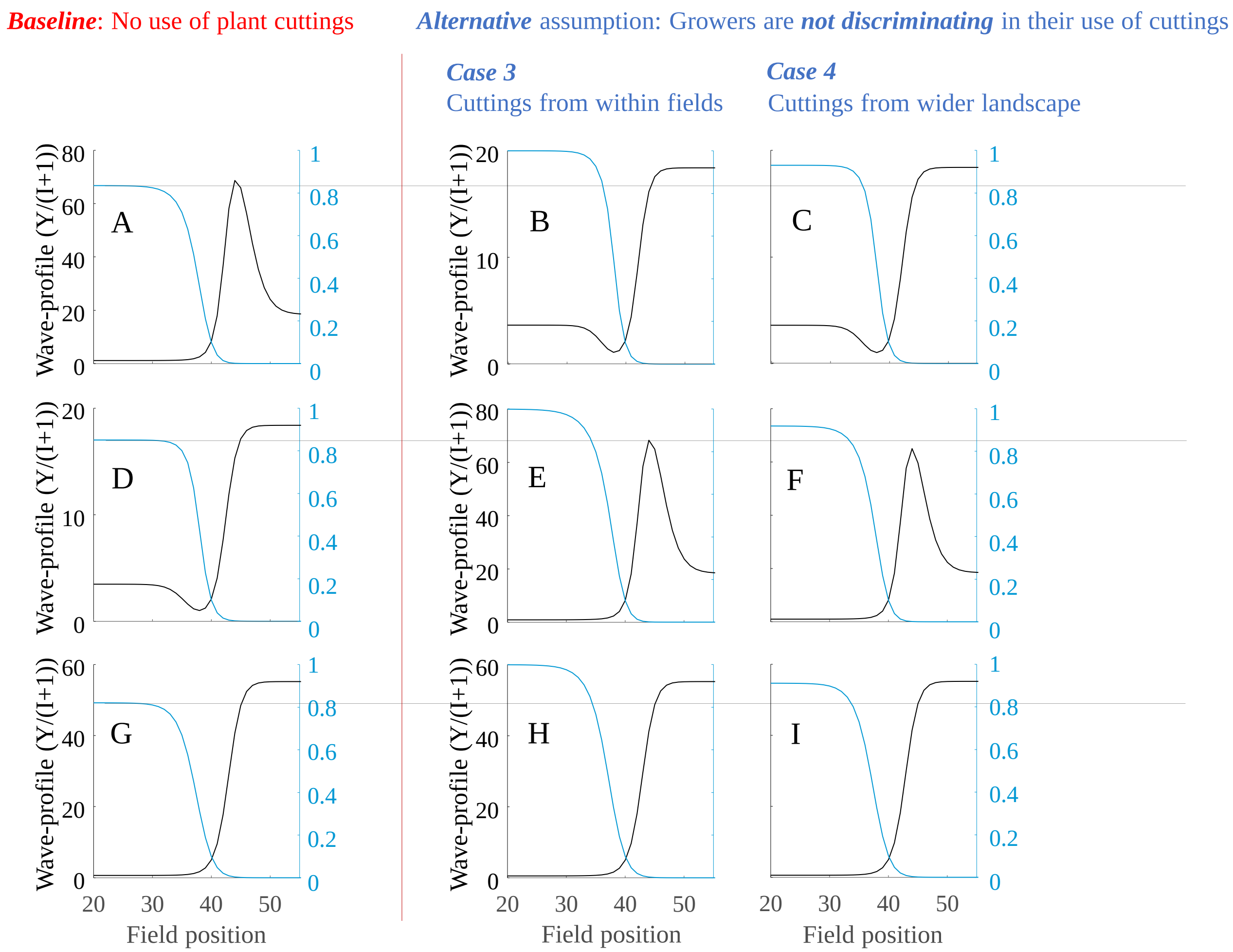

Figure S2.1: Pathogen emergence under three superabundance scenarios generate contrasting wave-profiles as identified in the main text (Fig. 2, Table 2) when propagation of the host crop involves potentially pathogen-exposed and pathogen-infected cuttings, i.e., where growers are not discriminating in their use of cuttings. Landscape simulation of insect vector and epidemiological dynamics when emergence is caused by: the arrival of a more fecund insect strain (A-C, INViS), by pathogen improvement of infected plant resource quality for insect vectors (D-F, PMiS), by enhanced environmental suitability for insect vectors across the landscape (G-I, EMiS). In A, D and G the results from Fig. 2 main text which correspond to no infected cuttings (equivalent to 100% clean seed, $pC = 1$ in Eq.s S2.2) are reproduced for comparison. In B-C, E-F and H-I where $pC = 0$, farmers are not discriminating in their use of infectious cuttings; in B, E and H the cuttings solely originate from the grower's field (case 3, system S2.2 with $cW = 1$); in C, F and I the cuttings originate from the wider landscape (case 4, system S2.2 with $cW = 0$). In A-F, black curves represent wave-profiles defined as the ratio of insect abundance to the number of infected plants within fields; light blue curves, for reference, represent disease fronts. The parameters were otherwise as described in caption of Fig. 2, main text. Snapshots were taken when the 40th field had reached 0.1 incidence. Simulations were run in MATLAB R2018a.

REFERENCES
