# Supplementary material for: The role of pathogen‐mediated insect superabundance in the East African emergence of a plant virus
Source: J Ecol. 2022 Mar 13;110(5):1113–24. doi: 10.1111/1365-2745.13854 (PMC9310957; doi:10.1111/1365-2745.13854)
Supplement: Supplementary file 3 — Supinfo3 [file JEC-110-1113-s001.pdf]

Donnelly and Gilligan The role of pathogen mediated insect superabundance in the east-African emergence of a plant virus

Supporting Information S3, Statistical analysis

We formulate a degree 2 polynomial mixed effects model of the wave-profile response variable (i.e., ratio of adult abundance to the number of infected plants +1 by field). The regression incorporates year of the response data and transect distance (i.e. for each field from the invaded side of the landscape transect) as fixed effects with distance having both linear and quadratic terms for each year. In addition, the mixed effects model accounts for random variation among fields (which leads to correlation in the response across observation years). The overall regression model is given by,

$$r_{ij} = \alpha_{0,i} + \alpha_{1,i} D_j + \alpha_{2,i} D_j^2 + f_j \tag{S3.1}$$

$$f_j \sim N(0, \sigma_{id}^2) \tag{S3.2}$$

$$y_{ij} \sim N(r_{ij}, \sigma_{res}^2) \tag{S3.3}$$

$$\text{where } \; y_{ij} = \frac{A_{ij}}{I_{ij} + 1}. \tag{S3.4}$$

where A_{ij} and I_{ij} in Eq. S3.4 represent adult vector abundance and the number of pathogen-infected plants in year i in field j respectively. In Eq. S3.1, D_j is the continuous transect distance variable: the distance from the invaded end of the transect, which is assumed to have been scaled, from km for field j, so that it occurs over the interval $[0, 1]$. The random effect term, f_j, accounts for random variation in field quality and is a consequence of repeated measures taken from each field (e.g., over the two years studied for the landscape experiment data, see main text), Eq. S3.2. The residual error is produced with variance σ_{res}^2, Eq. S3.3. Note that the intercept, $\alpha_{0,i}$, corresponds to the wave-profile

response at the invaded end of the landscape. Since in each year invasion is likely to be established at this location, the model may be simplified by the constraint $\alpha_{0,i}$=α_0 for all years i (see caption of Table 1, main text).

A Bayesian approach to model fitting is particularly suitable because a main parameter of interest, the turning point (denoted here by α'_λ for year λ), is compound in nature i.e., $\alpha'_\lambda = -\alpha_{1,i=\lambda}/\alpha_{2,i=\lambda}$. Whereas a posterior distribution for α' can be directly inferred using a Bayesian approach, inference of a confidence interval for α' through a frequentist approach is less straightforward [1].

The hypothesis test based upon model S3.1-S3.4 consists of two parts, a turning point test and a curvature test. In this paper, both turning point and curvature are assessed at the 95% significance level. The null hypothesis of a vector-orthodox wave-profile is rejected in year $i = \lambda$ if the wave-profile turning point is found to occur within the transect studied i.e., $0 < \alpha'_{i=\lambda}$ 95% $C.I. < 1$ (turning point test). In particular, the null hypothesis is rejected in favour of a vector-pushed hypothesis in year $i = \lambda$, if, in addition, $\alpha_{2,i=\lambda}$ 95% $CI > 0$ (curvature test)(i.e., internal minimum cf. Table S1.2). Alternatively, the null hypothesis is rejected in favour of vector-pulled if, in addition to the turning point condition, $\alpha_{2,i=\lambda}$ 95% $CI < 0$ (i.e., internal maximum cf. Table S1.2). In the Bayesian approach taken in this paper, hypothesis testing involves reporting the 2.5^{th} and 97.5^{th} percentile interval for the model parameters $\alpha_{2,i=\lambda}$ and $\alpha'_{i=\lambda}$: assessing if the former falls within the interval $[0, 1]$, and assessing if the latter is entirely positive or entirely negative (Table 1, main text).

REFERENCES

1. Plassmann F, and Khanna N. 2007 Assessing the precision of turning point estimates in polynomial regression functions. *Econometric Reviews*, 26(5), pp.503-528.
