# Supplementary material for: The role of pathogen‐mediated insect superabundance in the East African emergence of a plant virus
Source: J Ecol. 2022 Mar 13;110(5):1113–24. doi: 10.1111/1365-2745.13854 (PMC9310957; doi:10.1111/1365-2745.13854)
Supplement: Supplementary file 4 — Supinfo4 [file JEC-110-1113-s005.pdf]

Supporting Information S4, Analysis of Ugandan survey data 1992-1993

This supporting information details the methods involved in the analysis of a Ugandan landscape survey dataset [1] for regional spread of whitefly-borne severe cassava mosaic disease (henceforth, CMD).

Survey vs experimental landscape data

Both the experimental dataset [2] and the survey datasets [1] are based upon the same epidemic wave moving on an approximate north-south axis, but at different times/locations, and in addition the landscape survey dataset was conducted along two distinct transects ('central' and 'eastern' transects in Uganda [1]). The survey datasets [1] feature several additional factors that distinguish them from the experimental data [2], which we now outline.

- Whereas in the controlled conditions underlying the experimental data the incidence of infected cassava plants decreases relatively smoothly travelling southwards, in the survey data the incidence of infected cassava plants rises again at the southern most extremes of the transects (i.e., in advance of the wave-front). The authors [1] suggest that this is due to movement of infected cuttings to the urban areas that are found at the south of each transect (i.e., *Kampala* for the central transect and *Tororo* for the eastern transect). *For the application of our framework, we constrain our analysis to the monotonic part of the survey transects i.e. we omit the two*

most southerly locations from the survey for each transect on the assumption that enhanced movement of cassava around the cities introduces a different dynamic from the characteristic wave spread that is the focus of our analysis.

- Whereas the experimental data were longitudinal (the same fields were observed over two years), the survey data conducted at the same locales each season did not necessarily involve the exact same fields. Moreover, the survey data was collected over approximately a single year rather than the two years of the landscape experiment (July '92 - July '93, central transect; November '92 - August '93, eastern transect). *For the application of our framework to the survey data it was therefore necessary to omit the 'field' random effect from the survey data regressions.*

Wave-profile statistical analysis

The regression model underlying the hypothesis testing approach used in the main text and detailed in Supplementary Information 3, is modified to take account of the survey nature of the dataset, as indicated in the above section. As in Supplementary Information 3, we formulate a degree 2 polynomial model of the wave-profile response variable (i.e., ratio of adult abundance to the number of infected plants +1 by field). Whereas in Supplementary Information 3 the model incorporated random effects due to repeated measures in fields over multiple years, this is not suitable for the survey data which lacks repeated measures across years, as discussed in the above section. The overall regression model is given by,

$$r_j = \alpha_0 + \alpha_1 D_j + \alpha_2 D_j^2 \tag{S4.1}$$

$$y_j \sim N(r_j, \sigma^2_{res}) \tag{S4.2}$$

$$\text{where } \; y_j = \frac{\bar{A}_j}{\bar{I}_j + 1}. \tag{S4.3}$$

where the terms in Eq. S4.1 are no longer year specific - instead $\bar{A}_j$ and $\bar{I}_j$ representing field j adult abundance and the number of pathogen-infected plants, respectively, averaged over the months that were surveyed (which spanned approximately a single year). All parameters are otherwise as discussed in Supplementary Information 3. The hypothesis testing approach based upon model S4.1-S4.3 is otherwise as described in Supplementary Information 3.

REFERENCES

1. Legg JP, Ogwal S. 1998. Changes in the incidence of African cassava mosaic geminivirus and the abundance of its whitefly vector along south-north transects in Uganda. *Journal of Applied Entomology*, 122, 169-178.

2. Colvin J, *et al.* 2004 Dual begomovirus infections and high Bemisia tabaci populations drive the spread of a cassava mosaic disease pandemic. *Plant Pathology*, 53, 577-584.
