# Supplementary material for: The role of pathogen‐mediated insect superabundance in the East African emergence of a plant virus
Source: J Ecol. 2022 Mar 13;110(5):1113–24. doi: 10.1111/1365-2745.13854 (PMC9310957; doi:10.1111/1365-2745.13854)
Supplement: Supplementary file 5 — Supinfo5 [file JEC-110-1113-s004.pdf]

Supporting Information S5, Data sources

This supporting information details the references governing the choice of cassava mosaic disease parameter set used in the main text landscape simulation (Table S5.1). We also provide details on data digitisation, from a landscape experiment [1] and from a landscape survey [2], for the data analysis in the main text. Note that, in accordance with Colvin et al. [1], Legg and Ogwal [2] the epidemic of severe cassava mosaic disease in Uganda was assumed to be moving along an approximate north-south trajectory. Accordingly, distance of locations along transects, from the invaded end of the transect, were calculated with respect to a north-south axis. Note that the details make reference to Donnelly and Gilligan [3] - an online code repository in which datasets and dataset sources in the form of .ppt files can be found. Where filenames are referenced in the following sections they can be found in the above code repository.

Landscape experiment

Figure 1 of Colvin et al. [1] was converted into transect distances by measuring the distance along a north-south trajectory of transect locations ('DigitiseExpTransect.pptx', 'ExperimentOrientS.csv'). Figure 2 of Colvin et al. [1] was converted into insect abundance and disease incidence through digitisation of bar-charts ('DigitiseExpNumbers.pptx', 'ExpDataConversions.xlsx', 'ExperimentOrientS.csv').

Landscape survey

Figure 1 of Legg and Ogwal [2] was converted into transect distances by measuring the distance along a north-south trajectory of transect locations ('DigitiseSurveyTransect.pptx', 'SurveyCentralOrientS.csv', 'SurveyEasternOrientS.csv'). Figure 2 of Legg and Ogwal [2] was converted into insect abundance and disease incidence through digitisation of bar-charts ('DigitiseSurveyAbundance.pptx', 'DigitiseSurveyIncidence.pptx', 'DigitiseSurveyAvrNymphs.pptx', 'SurveyDataConversions.csv', 'SurveyCentralOrientS.csv', 'SurveyEasternOrientS.csv').

**Definition of parameter values used**	*Parameter values*	
Healthy plant mortality rate	$\mu=1/360$	0-0.025<br>(Holt et al., 1997)
Rate of additional removal of infected plants	$\delta=1/360$	Arbitrary<br>(set to c.monthly roguing)
Incubation rate of infection in plants	$\nu=1/30$	(Storey and Nichols, 1938)
Adult vector mortality rate	$b=1/50$	Set so that #insects per plant<300 in all scenarios
Adult dispersal rate	$\theta=1$	*Arbitrary*
Adult reproduction rate (for 0 vector abundance)	a=1*(100*b/2)	30-250 eggs per female<br>(Holt et al., 1997)
Adult reproduction limit (upper limit on density)	$K=40$	20-250<br>(Holt et al., 1997)
Nymph vector development rate	$\kappa=1/25$	19-29 days emergence<br>(Colvin et al. 2006)
Nymph vector mortality rate	$b_N=2b$	*Arbitrary*
Rate of acquisition of pathogen	$r^{acq}=0.032$	0.002-0.032<br>(Holt et al., 1997)
Rate of inoculation of pathogen	$r^{inoc}=r^{acq}$	0.002-0.032<br>(Holt et al., 1997)
Probability vector dispersal remains in field	$q = 0.5$	Arbitrary
Probability vector migration from field is lost	$u = 0.5$	Arbitrary

Table S5.1: Sources of parameter values used in the landscape simulation.

REFERENCES

1. Colvin J, *et al.* 2004 Dual begomovirus infections and high Bemisia tabaci populations drive the spread of a cassava mosaic disease pandemic. *Plant Pathology*, 53, 577-584.

2. Legg JP, Ogwal S. 1998. Changes in the incidence of African cassava mosaic geminivirus and the abundance of its whitefly vector along south-north transects in Uganda. *Journal of Applied Entomology*, 122, 169-178.

3. Donnelly, R. & Gilligan, C.A. (2020) Supporting code for *The role of pathogen mediated insect superabundance in the east-African emergence of a plant virus* Available at https://zenodo.org/badge/latestdoi/373580231

4. Holt J, Jeger MJ, Thresh JM, Otim-Nape GW. 1997 An epidemiological model incorporating vector population dynamics applied to African cassava mosaic virus disease. *Journal of Applied Ecology*, 1:793-806.

5. Storey HH, Nichols RF. 1938 Studies of the mosaic diseases of cassava. *Annals of Applied Biology*, 25(4):790-806.

6. Colvin, J., Omongo, C.A., Govindappa, M.R., Stevenson, P.C., Maruthi, M.N., Gibson, G., Seal, S.E., Muniyappa, V. 2006 Host-plant viral infection effects on arthropod-vector population growth, development and behaviour: management and epidemiological implications. *Advances in Virus Research*, 67, 419–452.
